# Early versus late oral feeding regimens following esophagectomy: a propensity score-matched observational cohort

**DOI:** 10.1093/dote/doaf068

**Published:** 2025-09-18

**Authors:** Cezanne D Kooij, Lucas Goense, B Feike Kingma, Robin B den Boer, Elles Steenhagen, Jelle P Ruurda, Richard van Hillegersberg

**Affiliations:** Department of Surgery, University Medical Center Utrecht, Utrecht, The Netherlands; Department of Surgery, University Medical Center Utrecht, Utrecht, The Netherlands; Department of Surgery, University Medical Center Utrecht, Utrecht, The Netherlands; Department of Surgery, University Medical Center Utrecht, Utrecht, The Netherlands; Department of Dietetics, University Medical Center Utrecht, Utrecht, The Netherlands; Department of Surgery, University Medical Center Utrecht, Utrecht, The Netherlands; Department of Surgery, University Medical Center Utrecht, Utrecht, The Netherlands

**Keywords:** esophagectomy, nutrition, survival

## Abstract

**Background:**

The optimal time to resume oral intake after esophagectomy remains debated, with practices varying across centers.

**Methods:**

This single-center, retrospective cohort study compared early and late oral feeding regimens after esophagectomy. Perioperative care was identical except for the feeding regimen. Early oral feeding began immediately post-surgery, while late oral feeding started on postoperative day 4 after swallow examination, with jejunostomy for early enteral tube feeding. Propensity score matching was used to reduce confounding. The primary outcome was overall survival. Secondary outcomes included complications, need for an alternative feeding route, hospital stay, readmission, and 90-day mortality.

**Results:**

Between May 2017 and October 2023, 406 patients underwent an esophagectomy (312 transthoracic; 94 transhiatal). After matching, 139 patients were included in both the early and late oral feeding groups. Overall complication rates did not significantly differ (84.9% vs. 77.7%; *P* = 0.124), but the late oral feeding group had less severe complications (48.9% vs. 36.7%; *P* = 0.039). The late oral feeding group showed lower leakage rates in intrathoracic anastomosis (33.3% vs. 13.3%; *P* = 0.008), but no differences for cervical anastomosis. The late oral feeding group had a shorter median hospital stay (12 vs. 11 days, *P* = 0.008). No differences in overall survival rates were found (Kaplan–Meier: *P* = 0.604, Cox regression: HR: 1.020, 95% CI 0.729–1.427, *P* = 0.907).

**Conclusions:**

Early and late oral feeding showed similar survival rates, but late oral feeding was associated with fewer severe complications, lower anastomotic leakage, and shorter hospital stay. Therefore, despite no survival difference, late oral feeding with jejunal feeding may lead to better postoperative outcomes.

## INTRODUCTION

Esophageal cancer is the seventh most common cancer worldwide, and the sixth leading cause of cancer-related mortality, with the majority of patients being diagnosed at an advanced stage.^1^ The cornerstone of curative treatment is esophagectomy with radical lymphadenectomy, often preceded by neoadjuvant therapy. This procedure is associated with significant morbidity and mortality, highlighting the importance of Enhanced Recovery After Surgery (ERAS) protocols. ERAS employs a multimodal approach, implementing perioperative measures to reduce complications and accelerate recovery.^2^

In esophageal cancer surgery, the postoperative feeding strategy is still topic of debate. While the importance of adequate feeding is widely acknowledged in order to optimize recovery, there is no clear consensus in literature concerning the timing of oral feeding and the methods of nutritional support.^3^ Traditionally, patients were kept nil-by-mouth after esophagectomy due to concerns about complications such as anastomotic leakage and aspiration pneumonia.^4^ However, evidence from other major gastrointestinal procedures such as colectomy shows no clear benefit to keeping patients nil-by-mouth.^5^ A multicenter randomized controlled trial compared early versus late oral feeding protocols in minimally invasive esophagectomy (MIE), demonstrating similar functional recovery and incidence and severity of postoperative complications.^6^ Additionally, a secondary analysis suggested a potential long-term survival benefit with early oral feeding, although the underlying mechanism of this association remains unclear.^7^

Consequently, the optimal timing for resuming oral feeding after esophagectomy remains uncertain, with practices varying across centers. To address this, the present study evaluates postoperative outcomes in matched cohorts of patients following either early or late oral feeding regimens, with a primary focus on the potential impact on long-term survival. Additionally, this study examines the association between feeding strategy and short-term outcomes including overall complications, complication severity, anastomotic leakage, need for an alternative feeding route, hospital, and intensive care unit (ICU) stay, readmission, and 90-day mortality.

## METHODS

### Study design and population

This observational retrospective cohort study included patients who underwent esophagectomy for esophageal cancer at the University Medical Center Utrecht, between May 2017 and October 2023. Procedures included transhiatal or transthoracic esophagectomy with gastric conduit reconstruction using an intrathoracic or cervical hand-sewn end-to-side anastomosis, which was the standard anastomotic technique in our center during the study period. Patients were excluded in case of absence of a gastric conduit, or if a stapled anastomosis and/or side-to-side or end-to-end approach was used, in order to reduce potential confounding and ensure a homogeneous study population with regard to surgical technique. Data were extracted from a prospectively maintained database. The institutional review board approved this study, and the requirement for informed consent was waived.

### Feeding regimens

The oral feeding regimen is part of the ERAS protocol. From May 2017 to February 2019, patients routinely followed an early oral feeding regimen, beginning oral intake immediately after surgery. On postoperative day (POD) 0 patients received a popsicle; on POD1–3, they were given a clear liquid diet and on POD4, they could expand to a full liquid diet, both including oral nutritional supplements. No formal assessment of gastric conduit emptying or swallowing function was performed prior to initiation of oral intake. Jejunostomy placement was not routinely performed during esophagectomy and was reserved for patients with poor preoperative nutritional status, defined as >10% weight loss within six months or >5% weight loss within a month. Patients were ideally discharged on oral feeding alone (full liquid diet for 2 weeks, followed by further expansion in consultation with a dietitian), although additional enteral tube feeding was provided if a jejunostomy had been placed. Although no uniform definition exists and the literature varies on what constitutes early oral feeding, our regimen aligns with what is generally considered early oral feeding.^8^

In March 2019, the protocol was revised to a late oral feeding regimen due to an increased incidence of delayed gastric emptying (10%) and 40% of patients needed an alternative feeding route due to lack of oral caloric intake. Under the revised protocol, patients routinely received an intraoperatively placed jejunostomy and enteral tube feeding was started at POD1 and gradually increased if bowel movements had resumed and there were no signs of ileus. Patients were kept nil-by-mouth from POD1–3, with sips of water introduced on POD4, following a satisfactory contrast swallow test. The routine contrast swallow test assessed gastric conduit emptying, as well as swallowing function (aspiration). After a satisfactory contrast swallow test, oral feeding could be increased with liquid diets. Patients were discharged with oral fluid diet for two weeks and maintained enteral tube feeding via the jejunostomy until adequate oral intake was achieved.

### Outcomes

The primary outcome was overall survival, defined as time from surgery to death or last follow-up. Secondary outcomes included postoperative complications, anastomotic leakage (clinically or radiologically, according to the Esophagectomy Complications Consensus Group [ECCG] classification), severity of anastomotic leakage (Clavien-Dindo ≥3a), need for an alternative feeding route (jejunostomy, nasojejunal feeding tube, and PICC line for total parenteral feeding [TPN]), hospital and ICU stay, readmissions, and 90-day mortality.

Anastomotic leakage was defined according to the ECCG as a full-thickness gastrointestinal defect involving esophagus, anastomosis, staple line, or conduit, regardless of presentation or method of identification.^9^ Anastomotic leakage was suspected when a patient had a combination of signs such as fever, tachycardia, elevated serum levels of C-reactive protein and/or elevated leucocytes. In such cases, patients underwent a computed tomography scan and an additional endoscopy if necessary. Upon diagnosis, patients were immediately placed on a nil-by-mouth regimen, received endoscopic placement of a nasogastric tube into the gastric conduit, and were started on intravenous antibiotics. Additional endoscopic and/or surgical interventions were applied depending on the severity of the leakage.^10^

All patient characteristics, peri- and postoperative outcomes were retrieved from a prospectively maintained database, with comprehensive registration of potential complications, including pulmonary, cardiac, urological, thromboembolic, neurological, wound-related, chyle-related, discussed and recorded during a weekly multidisciplinary team (MDT) meeting.

The Patient-Generated Subjective Global Assessment (PG-SGA) was used by trained dietitians to determine nutritional status and identify malnutrition two weeks after neoadjuvant therapy.^11^ If the post-neoadjuvant PG-SGA score was missing, the preoperative PG-SGA value was used if available.

### Statistics

To minimize the effect of possible confounders, propensity score matching was performed to compare the outcomes between the early and late oral feeding regimens. Possible confounders included gender, age, body mass index, American Society of Anesthesiology (ASA)-score, comorbidities, tumor location, cT-, cN-, and cM-stage, neoadjuvant therapy and surgical approach. Specifically, one-to-one propensity score matching was conducted with nearest-neighbor matching without replacement. The caliper width was adjusted to ensure that the matched cohort achieved standardized mean differences (SMDs) of <10% for all relevant confounders. This adjustment was made with the consideration of maintaining an optimal balance between achieving precise matching and preserving an adequate sample size, thereby avoiding an overly strict caliper that could reduce the number of matched pairs.^12^ This resulted in the formation of a cohort in which the two groups have comparable baseline characteristics. The baseline characteristics of the matched cohort and their SMDs are displayed in Table 1.

**Table 1:** Baseline characteristics

	Entire cohort			Matched cohort		
	Early feeding (*n* = 144)	Late feeding (*n* = 262)	SMD	Early feeding (*n* = 139)	Late feeding (*n* = 139)	SMD
Gender (male)	113 (78)	196 (75)	0.089	113 (81)	111 (80)	0.036
Age at time of operation in years (mean, SD)	66.1 (±9.1)	65.9 (±8.8)	0.0252	66.0 (±9.0)	66.3 (±9.4)	0.040
BMI in kg/m^2^ (mean, SD)	26.1 (±4.5)	25.5 (±4.6)	0.1175	25.6 (±4.5)	26.1 (±4.4)	0.099
ASA-score I II III IV	12 (8) 89 (62) 43 (30) 0 (0)	18 (7) 114 (44) 123 (47) 7 (3)	0.382	11 (8) 83 (60) 45 (32) 0 (0)	12 (9) 84 (60) 43 (31) 0 (0)	0.036
Comorbidities Overall Vascular Cardiac Pulmonary Diabetes	118 (82) 67 (57) 38 (32) 36 (31) 28 (24)	200 (76) 107 (54) 69 (35) 53 (27) 40 (21)	0.189 0.603 0.653 0.527 0.519	111 (80) 69 (50) 37 (27) 31 (22) 24 (17)	113 (81) 63 (45) 38 (27) 33 (24) 26 (19)	0.036 0.086 0.016 0.034 0.037
Tumor location Cervical Proximal Mid Distal GEJ	0 (0) 8 (6) 18 (13) 100 (69) 18 (13)	4 (2) 9 (3) 35 (13) 157 (60) 57 (22)	0.1150 0.0926 0.0260 0.2067 0.2799	0 (0) 6 (4) 22 (16) 94 (68) 17 (12)	0 (0) 5 (4) 18 (13) 98 (71) 18 (13)	0.000 0.021 0.062 0.081 0.036
Clinical T-stage cT1 cT2 cT3 cT4	8 (6) 23 (16) 102 (71) 11 (8)	9 (3) 52 (20) 164 (63) 37 (14)	0.102	8 (6) 18 (13) 97 (70) 16 (12)	7 (5) 23 (17) 98 (71) 11 (8)	0.086
Clinical N-stage cN0 cN+ cNx	56 (39) 87 (60) 1 (1)	124 (47) 136 (52) 2 (1)	0.164	56 (40) 82 (59) 1 (1)	56 (40) 82 (59) 1 (1)	0.000
Clinical M-stage cM0 cM1	141 (98) 3 (2)	254 (97) 8 (3)	0.067	136 (98) 3 (2)	136 (98) 3 (2)	0.0000
Neoadjuvant treatment No neoadjuvant treatment Chemotherapy Radiotherapy Chemoradiotherapy	16 (11) 6 (4) 0 (0) 122 (85)	32 (12) 39 (15) 1 (0) 190 (73)	0.035 0.536 0.000 0.328	13 (9) 8 (6) 0 (0) 118 (85)	16 (12) 6 (4) 0 (0) 117 (84)	0.070 0.065 0.000 0.019
Approach TH (cervical anastomosis) TT (cervical anastomosis) TT (intrathoracic anastomosis)	29 (20) 48 (33) 67 (47)	65 (25) 83 (32) 114 (44)	0.116 0.035 0.060	29 (21) 50 (36) 60 (43)	28 (20) 45 (32) 66 (48)	0.017 0.075 0.086
Open vs. MIE Open MIE	2 (1) 142 (99)	10 (4) 252 (96)	0.206 0.206	2 (1) 137 (99)	2 (1) 137 (99)	0.000 0.000

Abbreviations: ASA, American Society of Anesthesiology; GEJ, gastro-esophageal junction, MIE, minimally invasive esophagectomy; SMD, standardized mean difference; SD, standard deviation; TH, transhiatal; TT, transthoracic.

Analyses were performed according to the intention-to-treat principle. Overall survival was compared between the matched cohorts using Kaplan–Meier curves with log-rank tests. Additionally, a multivariable Cox regression analysis on the full (unmatched) cohort assessed the impact of the feeding regimen, reporting the hazard ratio (HR) with 95% confidence interval (CI) while adjusting for the following confounding factors: age, gender, ASA-score, comorbidity, neoadjuvant therapy, open/MIE, surgical approach, radicality, histology, pT-stage, pN-stage, and severe complications.

Categorical data were analyzed with Chi-square tests, continuous data with normal distributions were compared using independent samples *t*-tests and those with non-normal distributions using the Mann–Whitney *U* test. Analyses were performed using SPSS version 29.0, and R 3.1.2 open-source software with MatchIt and optmatch, packages (http://www.R-project.org). *P* < 0.050 was considered statistically significant.

## RESULTS

Between May 2017 and October 2023, 412 patients underwent an esophagectomy. Of these, 6 patients were excluded from the analysis because no gastric conduit was created (*n* = 3) or the anastomosis was performed in another fashion than a (robotic) hand-sewn end-to-side technique (*n* = 3, 1 mechanical and 2 end-to-end anastomoses). The included patients were categorized into two groups based on the oral feeding regimen: early oral feeding (*n* = 144) and late oral feeding (*n* = 262). The SMDs of the baseline characteristics between groups are depicted in Table 1. After propensity score matching, 139 patients from each group were matched, resulting in smaller SMDs and ensuring that the groups had comparable baseline characteristics (Table 1).

### Patient and tumor characteristics

The matched cohorts (*n* = 278) were predominantly male (*n* = 224, 81%), with a mean age of 66 years (Table 1). Most had comorbidities (*n* = 224, 81%), primarily vascular (*n* = 132, 48%). Tumors were mainly located in the distal esophagus (*n* = 192, 69%), with cT3 as the most common stage (*n* = 195, 70%). Most patients received neoadjuvant therapy (*n* = 249, 90%), mostly chemoradiotherapy (*n* = 235, 85%). Surgical approaches included transthoracic esophagectomy with intrathoracic (*n* = 126, 45%) or cervical anastomosis (*n* = 95, 34%), and transhiatal esophagectomy (*n* = 57, 21%). Almost all procedures were minimally invasive (*n* = 274, 99%). Table 2 displays PG-SGA assessments; nutritional status after neoadjuvant therapy was similar between the groups (*P* = 0.753).

**Table 2:** Patient-Generated Subjective Global Assessment (PG-SGA)^‡^

	Early feeding (*n* = 139)	Late feeding (*n* = 139)	*P*-value^*^
A—Well nourished	75 (54.0%)	78 (56.1%)	0.753
B—Moderately malnourished/suspected malnutrition	35 (25.2%)	36 (25.9%)	
C—Severely malnourished	9 (6.5%)	7 (5.0%)	
Missing	20 (14.4%)	18 (12.9%)	

Abbreviation: PG-SGA, patient-generated subjective global assessment.

^*^ *P*-values <0.05 are considered statistically significant. Data were assessed using a Mann–Whitney *U* test.

### Outcomes

**Figure 1:**
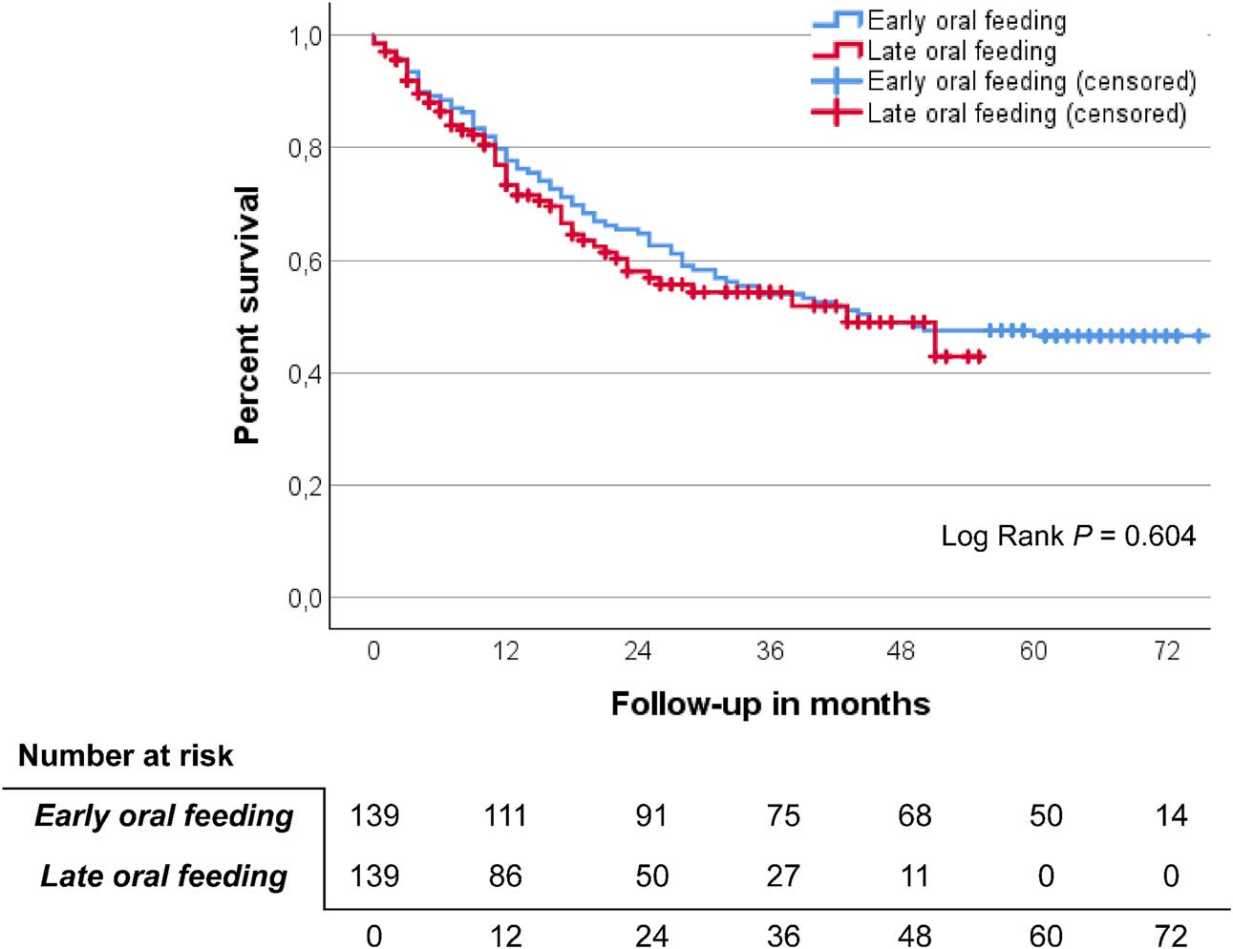
Kaplan–Meier curves of the overall survival of the early oral feeding and late oral feeding group (log rank *P* = 0.604).

In the matched cohorts, the median follow-up was 45 months (IQR 15–64 months) for patients in the early oral feeding group and 16 months (IQR 7–32 months) for patients in the late oral feeding group. The primary outcome measure, overall survival, did not differ between the matched groups, as shown in the Kaplan–Meier curve in Fig. 1 (log rank *P* = 0.604). Similarly, the multivariable Cox regression analysis of the entire unmatched cohort demonstrated no significant difference in survival between the feedings regimens, with an HR of 1.020 (95% CI 0.729–1.427, *P* = 0.907; Table 3).

**Table 3:** Multivariable analysis (Cox regression) on the impact of the feeding regimen and confounding factors on overall survival

		HR (95% CI)	*P*-value^*^
Age	Continuous	1.010 (0.992–1.029)	0.285
Gender	Male/female	0.959 (0.654–1.407)	0.832
ASA-score	ASA1–2/ASA3–4	1.530 (1.108–2.113)	0.010
Comorbidities	No/yes	0.981 (0.653–1.473)	0.927
Neoadjuvant therapy	No/yes	1.121 (0.690–1.823)	0.654
Open/MIE	Open/MIE	1.208 (0.464–3.147)	0.698
Surgical approach	Transthoracic/transhiatal	0.913 (0.620–1.344)	0.645
Radicality	R0/R1–2	3.268 (2.026–5.271)	<0.001
Histology	AC/SCC	0.983 (0.664–1.493)	0.983
pT-stage	pT0–1/pT2–4	1.755 (1.210–2.546)	0.003
pN-stage	pN0/pN+	2.356 (1.683–3.296)	<0.001
Complication≥CD3a	No/yes	1.462 (1.070–1.997)	0.017
Feeding regimen	Early/late oral feeding	1.020 (0.729–1.427)	0.907

Abbreviations: ASA, American Society of Anesthesiology; AC, adenocarcinoma; CI, confidence interval; CD, Clavien-Dindo; HR, hazard ratio; MIE, minimally invasive esophagectomy; SCC, squamous cell carcinoma.

^*^ *P*-values <0.05 are considered statistically significant.

As shown in Table 4, the overall postoperative complication rate did not differ between the early and late oral feeding groups (84.9% vs. 77.7%; *P* = 0.124). However, the incidence of severe complications did demonstrate a significant difference (48.9% vs. 36.7%, *P* = 0.039). Length of hospital stay was shorter in the late oral feeding group (12 days vs. 11 days; *P* = 0.008). There were no significant differences between the groups in terms of ICU length of stay, readmission rates, or 90-day mortality.

**Table 4:** Postoperative outcomes

	Early feeding (*n* = 139)	Late feeding (*n* = 139)	*P*-value^*^
Postoperative complication(s) Overall complications	118 (84.9%)	108 (77.7%)	0.124
Complications ≥Clavien-Dindo 3a Anastomotic leakage Pneumonia Chyle leakage	68 (48.9%) 40 (28.8%) 47 (33.8%) 15 (10.8%)	51 (36.7%) 26 (18.7%) 46 (33.1%) 9 (6.5%)	0.039 0.048 0.899 0.200
Length of hospital stay (median, IQR)	12 (9–21)	11 (8–15)	0.008
Length of ICU stay (median, IQR)	1 (1–3)	1 (1–2)	0.087
Readmission	23 (16.5%)	25 (18.0%)	0.751
Follow-up Mortality <90 days postoperatively	6 (4.3%)	6 (4.3%)	1.000

Abbreviations: IQR, interquartile range.

^*^ *P*-values <0.05 are considered statistically significant. Data were assessed using a Chi-Square test or an independent samples *t*-test.

The occurrence of anastomotic leakage was significantly higher in the early oral feeding group (*n* = 40, 28.8%) compared to the late oral feeding group (*n* = 26, 18.7%) (*P* = 0.048) (Table 4). Additionally, the early oral feeding group had a significantly higher incidence of severe anastomotic leakage (Clavien-Dindo ≥3a) compared to the late oral feeding group (25.2% vs. 13.7%; *P* = 0.015). However, there were no significant differences in the grade of anastomotic leakage according to the ECCG between the groups (*P* = 0.402). In a subgroup analysis (Supplementary Table S1), comparing early and late oral feeding groups across different surgical approaches, a significant difference in incidence and severity of anastomotic leakage was observed only for the intrathoracic anastomosis: 33.3% vs. 13.3% (*P* = 0.008) for incidence, and 30.3% vs. 11.4% (*P* = 0.011) for severity, respectively. No significant differences in anastomotic leakage rates were found for patients with a cervical anastomosis.

Table 5 shows that an intraoperative jejunostomy was placed in 42% of patients (*n* = 59) in the early oral feeding group and 98% of patients (*n* = 136) in the late oral feeding group. An alternative feeding route was required for 35.3% of patients (*n* = 49) in the early oral feeding group and 10.1% (*n* = 14) in the late oral feeding group. In the early oral feeding group, the alternative feeding route primarily involved placement of a PICC line for TPN (*n* = 17, 12.2%), placement of a nasojejunal feeding tube (*n* = 11, 7.9%), or both (*n* = 12, 8.6%). In the late oral feeding group, a PICC line for TPN was used in 6.5% of patients (*n* = 9).

**Table 5:** Postoperative feeding routes

	Early feeding (*n* = 139)	Late feeding (*n* = 139)
Jejunostomy during esophagectomy	59 (42.4%)	136 (97.8%)
Alternative feeding route	49 (35.3%)	14 (10.1%)
Jejunostomy Jejunostomy + nasojejunal feeding tube Jejunostomy + PICC line (TPN) PICC line (TPN) Nasojejunal feeding tube PICC line (TPN) + nasojejunal feeding tube	4 (2.9%) 1 (0.7%) 4 (2.9%) 17 (12.2%) 11 (7.9%) 12 (8.6%)	0 (0.0%) 2 (0.7%) 0 (0.0%) 9 (6.5%) 3 (2.2%) 0 (0.0%)

Abbreviations: PICC, peripherally inserted central catheter; TPN, total parenteral nutrition.

Among patients with an intraoperative jejunostomy (*n* = 195), 9% (*n* = 5) in the early, and 8% (*n* = 11) in the late oral feeding group required an alternative feeding route. Half of these cases (*n* = 8, 4.1%) were due to jejunostomy complications, including displacement (*n* = 3, 1.5%), wound abscess (*n* = 3, 1.5%), and obstruction (*n* = 2, 1.0%). The remaining patients (*n* = 8, 4.1%) required an alternative feeding route due to chyle leakage (*n* = 5, 2.6%), delayed bowel activity (*n* = 1, 0.5%), peritonitis/jejunum perforation (*n* = 1, 0.5%), and respiratory insufficiency/gastric conduit stasis (*n* = 1, 0.5%).

## DISCUSSION

This propensity score-matched observational cohort study showed no differences in overall survival after esophagectomy between patients who followed an early oral feeding regimen and those who followed a late oral feeding regimen. However, late oral feeding was associated with lower rates of severe complications and anastomotic leakage among patients who underwent esophagectomy with intrathoracic anastomosis. Additionally, the length of hospital stay was shorter in the late oral feeding group. Other postoperative outcomes did not show significant differences between the groups.

This study used overall survival as the primary outcome to assess the timing of oral intake. This interest arose from a side-study of the NUTRIENT II trial, comparing early (*n* = 63) and late (*n* = 66) oral feeding protocols following esophagectomy.^7^ The results showed Kaplan–Meier curves favoring early oral feeding for both 5-year overall (61.9% vs. 46.2%, log rank *P* = 0.115) and disease-free survival (52.9% vs. 33.3%, log rank *P* = 0.047). All patients in the cohort died from metastatic disease of the primary esophageal cancer, except for one patient in each group who died from non-cancer-related causes. The (severity of) complications did not seem to influence overall survival. Although the authors noted that the reason behind the possible correlation was not fully understood, baseline characteristics indicated that comorbidities (63.5% vs. 72.7%, *P* = 0.260), cT-stage (III: 46.0% vs. 50.8%, *P* = 0.787), pT-stage (pT3: 28.6% vs. 40.9%, *P* = 0.202), and pN-stage (pN2–3: 11.1% vs. 18.2%, *P* = 0.102) were more favorable in the early oral feeding group, which may explain the survival benefit related to oncological progression.^7^ Other studies, mostly retrospective, have found no clear advantage of one feeding regimen over the other, which aligns with our findings.^13^

Concerns regarding anastomotic leakage has been a major factor in delaying the resumption of oral intake following esophagectomy, given its association with morbidity, extended hospital stay, and increased mortality risk.^8^ Swallowing a bolus may increase tension on the anastomosis, while gastric distension after oral intake could further increase the risk of anastomotic leakage. It has been hypothesized that delaying oral intake may allow healing before stressing the anastomosis, thereby reducing the risk of leakage.^14^ In our study, early oral feeding increased leakage in patients with an intrathoracic anastomosis. Remarkably, this effect was not observed in patients with a cervical anastomosis. This may be due to the physiological benefit of reduced tension on the anastomosis immediately postoperatively in the late oral feeding protocol, allowing more time for healing. Additionally, the intrathoracic anastomosis might be more exposed to traction forces from feeding, whereas the cervical anastomosis is more fixed due to its passage through the thoracic inlet. Of note, the observed higher proportion of severe (Clavien-Dindo grade ≥ 3a) complications in the early oral feeding group (48.9% vs. 36.7%, *P* = 0.039) can largely be attributed to the significantly increased rate of severe anastomotic leakage (25.2% vs. 13.7%, *P* = 0.015).

While some studies suggest that late oral feeding reduces anastomotic leakage, other evidence indicates that early oral feeding does not increase rates of complications, including anastomotic leakage.^14^^,^^15^ However, evidence is limited by inconsistent definitions, selection bias, variations in nutritional support, and unaccounted confounders. Larger, high-quality studies are needed to evaluate impact of confounding variables like surgical approach, feeding routes, nutritional support methods, and diet progression.^16^ Therefore, consistent with previous literature, our study demonstrated that both early and late oral feeding regimens are comparable in terms of overall complications, suggesting that both approaches are safe. However, late oral feeding may offer a potential advantage in reducing anastomotic leakage rates, particularly in intrathoracic anastomoses.

Esophageal cancer patients are particularly vulnerable to malnutrition, with over 70% experiencing significant weight loss at diagnosis due to dysphagia and cachexia.^17^ Surgery further complicates nutritional intake through reduced stomach volume, early satiety, and postoperative complications.^17^^,^^18^ As such, early nutritional intervention and the involvement of dietetic professionals to address malnutrition are essential.^18^^,^^19^ Additionally, although the optimal timing for oral intake initiation remains debated, enteral nutrition is crucial for preventing postoperative nutritional decline.^16^ By placing a jejunostomy intraoperatively, as is routine in the late oral feeding protocol, enteral nutrition can be provided via enteral tube feeding irrespective of the oral intake. With experience of both feeding regimens in our clinical practice, we noted that routine jejunostomy placement improves comfort of patients during the period of postoperative feeding. In the early oral feeding cohort, a jejunostomy was not routinely placed, yet 35% of patients needed an alternative feeding route due to insufficient caloric intake following esophagectomy. This adds stress for patients and burdens health care providers. In fact, eating with a gastric conduit and a vagotomy requires adaption, which enteral tube feeding via jejunostomy facilitates by easing the transition to full oral intake. While it cannot fully meet caloric needs immediately post-surgery, it provides controlled support as recovery progresses. In the Netherlands, most centers routinely place a jejunostomy, with minimal additional risk and low complication rates in centers with experience in its (routine) placement.^20^

Strengths of this study include its relatively large cohort, a Western-representative esophageal cancer population, and comprehensive data from a prospectively maintained database with weekly MDT-verified complications. However, there are some limitations. Despite being representative, the cohort is heterogeneous. To address this, propensity score matching was performed. Additionally, follow-up varied between groups due to changes in oral feeding protocols over time, with shorter follow-up for late oral feeding. Lastly, temporal bias should be considered, as esophagectomy with intrathoracic anastomosis became routine practice in 2017, likely improving outcomes over time, potentially favoring the late oral feeding cohort.

In conclusion, this study found no differences in overall survival between early and late oral feeding cohorts after esophagectomy, suggesting that both approaches are safe with regard to survival. However, late oral feeding with early enteral tube feeding was associated with lower severe complication rates, reduced anastomotic leakage in esophagectomy with intrathoracic anastomosis, and a shorter hospital stay. Other outcomes were similar between groups. These findings indicate that there is no strong evidence to support the need for immediate oral intake resumption. Instead, despite no difference in survival, late oral feeding including routine jejunostomy may be preferable due to its association with better postoperative outcomes and a reduced need for an alternative feeding route.

## Supplementary Material

Early_versus_late_feeding_Supplementary_Material_04032025_doaf068

## Notes

Specific author contributions: Cezanne Kooij (Conceptualization, Formal analysis, Methodology, Writing—original draft, Writing—review & editing), Lucas Goense (Conceptualization, Formal analysis, Methodology, Supervision, Writing—original draft, Writing—review & editing), B. Feike Feike Kingma (Conceptualization, Methodology, Supervision, Writing—original draft, Writing—review & editing), Robin B. den Boer (Conceptualization, Methodology, Writing—review & editing), Elles Steenhagen (Conceptualization, Writing—review & editing), Jelle P. Ruurda (Conceptualization, Methodology, Supervision, Writing—review & editing), Richard van Hillegersberg (Conceptualization, Methodology, Supervision, Writing—review & editing).

Financial support: No funding was requested nor received for the current study.
