# Supplementary material for: Early versus late oral feeding regimens following esophagectomy: a propensity score-matched observational cohort
Source: Dis Esophagus. 2025 Sep 18;38(5):doaf068. doi: 10.1093/dote/doaf068 (PMC12490072; doi:10.1093/dote/doaf068)
Supplement: Early_versus_late_feeding_Supplementary_Material_04032025_doaf068 [file early_versus_late_feeding_supplementary_material_04032025_doaf068.docx]

**Supplementary Table 1: Anastomotic technique and anastomotic leakage in the total matched cohort and per surgical approach**

|  | **Early feeding** | **Late feeding** | **p-value^*^** |
| --- | --- | --- | --- |
| Total | **n=139** | **n=139** |  |
| Anastomotic leakage | 40 (28.8%) | 26 (18.7%) | **0.048** |
| Clavien Dindo ≥ 3a | 35 (25.2%) | 19 (13.7%) | **0.015** |
| Grade of AL (according to ECCG)  Grade 1  Grade 2  Grade 3 | 4 (2.9%)  16 (11.5%)  20 (14.4%) | 6 (4.3%)  9 (6.5%)  12 (8.6%) | 0.402 |
| Transhiatal esophagectomy | **n=28** | **n=29** |  |
| Anastomotic leakage | 6 (21.4%) | 3 (10.3%) | 0.251 |
| Clavien Dindo ≥ 3a | 4 (14.3%) | 2 (6.9%) | 0.363 |
| Grade of AL (according to ECCG)  Grade 1  Grade 2  Grade 3 | 1 (3.6%)  3 (10.7%)  2 (7.1%) | 1 (3.4%)  2 (6.9%)  1 (3.4%) | 0.728 |
| Ivor Lewis esophagectomy | **n=66** | **n=60** |  |
| Anastomotic leakage | 22 (33.3%) | 8 (13.3%) | **0.008** |
| Clavien Dindo ≥ 3a | 20 (30.3%) | 7 (11.7%) | **0.011** |
| Grade of AL (according to ECCG)  Grade 1  Grade 2  Grade 3 | 2 (3.0%)  12 (18.2%)  8 (12.1%) | 1 (1.7%)  4 (6.7%)  3 (5.0%) | 0.958 |
| McKeown esophagectomy | **n=45** | **n=50** |  |
| Anastomotic leakage | 12 (26.7%) | 15 (30.0%) | 0.719 |
| Clavien Dindo ≥ 3a | 11 (24.4%) | 10 (20.0%) | 0.602 |
| Grade of AL (according to ECCG)  Grade 1  Grade 2  Grade 3 | 1 (2.2%)  1 (2.2%)  10 (35.7%) | 4 (8.0%)  3 (6.0%)  8 (16.0%) | 0.108 |

Abbreviations: ECCG = Esophagectomy Complications Consensus Group.

^*^ p-values < 0.05 are considered statistically significant. Categorical data were assessed using the Chi-Square test, ordinal variables were analyzed using a Mann Whitney U test.
